# A Comparative Study: Cardioprotective Effects of High-Intensity Interval Training Versus Ischaemic Preconditioning in Rat Myocardial Ischaemia–Reperfusion

**DOI:** 10.3390/life14030310

**Published:** 2024-02-27

**Authors:** Jia-Yuan Zhang, Szu-Kai Fu, Hsia-Ling Tai, Kuo-Wei Tseng, Chia-Yu Tang, Chia-Hsien Yu, Chang-Chi Lai

**Affiliations:** 1 Graduate Institute of Sports Training, University of Taipei, Taipei 11153, Taiwan; d10635004@go.utaipei.edu.tw (J.-Y.Z.); danatai1008@go.utaipei.edu.tw (H.-L.T.); jimmy1130201@utaipei.edu.tw (C.-Y.T.); d10835005@go.utaipei.edu.tw (C.-H.Y.); 2 Department of Exercise and Health Sciences, University of Taipei, Taipei 11153, Taiwan; skyfu218@utaipei.edu.tw (S.-K.F.); fossil@utaipei.edu.tw (K.-W.T.)

**Keywords:** high-intensity interval training, ischemic preconditioning, myocardial ischemia and reperfusion injury, coronary artery occlusion, apoptosis

## Abstract

(1) Background: Years of research have identified ischemic preconditioning (IPC) as a crucial endogenous protective mechanism against myocardial ischemia–reperfusion injury, enhancing the myocardial cell’s tolerance to subsequent ischemic damage. High-intensity interval training (HIIT) is promoted by athletes because it reduces exercise duration and improves metabolic response and cardiopulmonary function. Our objective was to evaluate and compare whether HIIT and IPC could reduce myocardial ischemia and reperfusion injury in rats. (2) Methods: Male Sprague-Dawley rats were divided into four groups: sham surgery, coronary artery occlusion (CAO), high-intensity interval training (HIIT), and ischemic preconditioning (IPC). The CAO, HIIT, and IPC groups experienced 40 min of coronary artery occlusion followed by 3 h of reperfusion to induce myocardial ischemia–reperfusion injury. Subsequently, the rats were sacrificed, and blood samples along with cardiac tissues were examined. The HIIT group received 4 weeks of training before surgery, and the IPC group underwent preconditioning before the ischemia–reperfusion procedure. (3) Results: The HIIT and IPC interventions significantly reduced the extent of the myocardial infarction size and the levels of serum troponin I and lactate dehydrogenase. Through these two interventions, serum pro-inflammatory cytokines, including TNF-α, IL-1β, and IL-6, were significantly decreased, while the anti-inflammatory cytokine IL-10 was increased. Furthermore, the expression of pro-apoptotic proteins PTEN, caspase-3, TNF-α, and Bax in the myocardium was reduced, and the expression of anti-apoptotic B-cell lymphoma 2 (Bcl-2) was increased, ultimately reducing cellular apoptosis in the myocardium. In conclusion, both HIIT and IPC demonstrated effective strategies with potential for mitigating myocardial ischemia–reperfusion injury for the heart.

## 1. Introduction

In the realm of medicine, cardiovascular diseases (CVDs) persist as one of the primary causes of mortality and morbidity worldwide. Among them, acute myocardial infarction (MI) stands as a principal culprit within the spectrum of cardiovascular disorders [1]. During the treatment of acute MI, it is common to observe myocardial ischemia, characterized by reduced perfusion, leading to compromised metabolism and irregular cardiac function [2]. In particular, myocardial ischemia and reperfusion injury (MIRI) is a phenomenon in which tissue damage occurs due to calcium overload, leading to mitochondrial abnormalities, increased free radicals, high ATP consumption, myocardial energy metabolism defects, and inflammation, which causes the release of apoptotic factors upon the restoration of blood flow after myocardial ischemia [3]. MIRI not only harms the myocardium but also triggers systemic inflammatory responses, including the production of proinflammatory cytokines such as TNF-α, IL-1β, and IL-6, leading to necrosis and apoptosis [4]. Mitochondrial dysfunction during MIRI leads to the release of cytochrome C into the cytoplasm, activating caspase-3-mediated apoptosis [5]. However, cytochrome c leakage can be inhibited by Bcl-2/Bcl-xL [6]. TNF-α exacerbates MIRI during the early reperfusion stages, activating NF-κB, promoting leukocyte infiltration, and inducing apoptosis [7]. Exercise training is a cost-effective and safe strategy for preventing and treating cardiovascular diseases [8]. The predominant reason for the lack of physical activity in modern individuals is often attributed to “insufficient time” [9]. During such circumstances, high-intensity interval training (HIIT) emerges as a highly recommended exercise modality. HIIT involves repeated, short bouts of high-intensity exercise (≥90% HRmax) interspersed with periods of rest or low-intensity exercise; it may overcome time limitations related to exercise participation [10,11]. Previous studies have indicated that HIIT is more effective than low–medium intensity exercises in improving cardiovascular risk factors, reducing the risk of cardiovascular death, and alleviating cardiomyopathy, diabetes-induced obesity, and pathological myocardial hypertrophy in rats with heart failure after MI [12,13,14,15]. Currently, there are numerous methods to decrease MIRI in animal models, including postischemic physiological adaptation and advanced administration of protective drugs. Ischemic preconditioning (IPC) involves exposure to mild ischemia–reperfusion, reducing tissue injury by helping the body adapt to ischemic conditions [16]. IPC reduces cellular apoptosis, improves cardiac metabolism, and lowers the risk of coronary artery disease and heart failure. This is achieved by inhibiting inflammatory cell activation, regulating anti-apoptotic and pro-apoptotic proteins, and activating protein kinase C [17,18]. Furthermore, IPC stimulation induces the release of various secretions such as acetylcholine and adenosine from myocardial cells, initiating cardiac protective signaling pathways including the activation of proteins such as Protein Kinase B (Akt) and Extracellular Signal-Regulated Kinases 1 and 2 (Erk1/2). Subsequently, these protein kinases activate transcription factors such as Activator Protein 1 (AP-1) and Hypoxia-Inducible Factor 1 alpha (HIF-1α), thereby promoting the expression of prostaglandin G/H synthase (COX-2) and heat shock proteins, inducing cardiac protection. Moreover, studies have demonstrated IPC’s ability to protect mitochondrial function, reduce calcium overload, minimize reactive oxygen species (ROS) production, and inhibit mitochondrial permeability transition pore (MPTP) opening, all of which constitute integral mechanisms of cardiac protection strategies [19].

Myocardial ischemia and reperfusion—pathophysiological processes that accompany acute MI and cardiac surgery—are associated with significant risks of mortality and morbidity, prolonged hospitalization, and increased healthcare expenses. In this study, we hypothesize that HIIT, similar to IPC, reduces myocardial damage following ischemia–reperfusion by inhibiting inflammatory cell activation and modulating apoptotic signal transduction. We concurrently assessed the effects of both HIIT and IPC on myocardial damage and apoptotic signaling proteins in rat hearts post-ischemia–reperfusion. This aims to clarify their similarities and differences in mitigating damage from ischemia–reperfusion and their roles in preventive medicine for overall human health.

## 2. Materials and Methods

### 2.1. Ethics Approval

This study was approved by an institution affiliated with one of the authors (Taipei, Taiwan; IACUC approval no. UT109002). Notably, all the animals in the study were treated humanely in accordance with the Guide for the Care and Use of Laboratory Animals [20].

### 2.2. Animal Preparation

**Figure 1:**
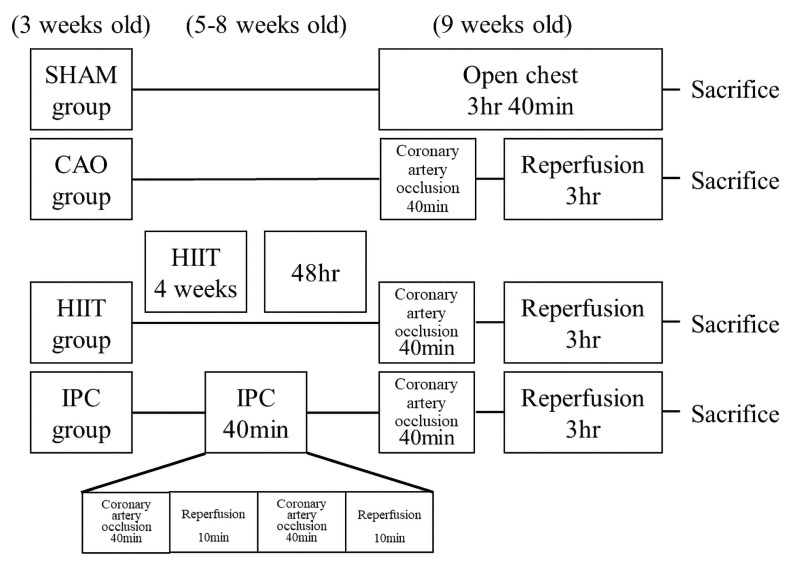
Study design of the experiment. All rats except for those in the sham group underwent a 40 min coronary artery occlusion and 3 h reperfusion after receiving various treatments. The sham group did not receive any pretreatment during the treatment period. Before the experiment, the HIIT group underwent 4 weeks of high-intensity interval training. The IPC group underwent ischemic preconditioning through two 10 min episodes of coronary artery occlusion and 10 min reperfusion. HIIT, high–intensity interval training; IPC, ischemic preconditioning.

Forty three-week-old male Sprague-Dawley (SD) rats were procured from BioLASCO. The rats had access to food ad libitum. The room temperature was maintained at 25 °C, and humidity was maintained at 55 ± 5%. The light cycle was set from 6:00 AM to 6:00 PM. The rats were randomly classified into the following four groups: the sham (n = 10), coronary artery occlusion (CAO, n = 10), HIIT (n = 10), and ischemic precondition (IPC, n = 10) groups. All groups of rats were sacrificed at the age of 9 weeks. In the sham group, rats were anesthetized and open thoracic surgery was performed. After 220 min, the rats were sacrificed. In the CAO group, rats were anesthetized, and the proximal left anterior descending coronary artery was ligated for 40 min, followed by reperfusion for 180 min. Subsequently, the rats were sacrificed. The HIIT group of rats commenced adaptation training at the age of 3 weeks. Initial training involved treadmill exercises using the T510 treadmill (Diagnostic & Research Instruments, Singa, Taiwan) at a speed of 20 m/min with a 5-degree incline, lasting for 30 min each day. By the 4th week of age, the treadmill speed was adjusted to 20 m/min with a 10-degree incline, maintaining a daily exercise duration of 30 min. From the 5th to the 8th week of age, rats entered the formal training period. Prior to each training session, a 5 min warm-up at a speed of 15 m/min was conducted on the treadmill. The training then commenced with the treadmill set at a speed of 50 m/min and a 10-degree incline, lasting for 30 min each day (2 min of rest followed by 1 min of sprinting repeated 10 times) (≈85 VO_2_max) [21]. This training regimen was implemented 4 days per week for a continuous duration of 4 weeks. Training was started at 6:30 PM and performed five times per week. When necessary, a mild electric current was gently applied to the posterior body of the rats to encourage compliance and ensure adherence to the training protocol. The training protocol for the HIIT group of rats is outlined in Table 1. In the IPC group, before coronary artery occlusion intervention, the rats underwent partial occlusion of the left anterior descending artery for 10 min, followed by reperfusion for 10 min. This cycle was repeated twice. Subsequently, partial occlusion of the left anterior descending artery for 40 min was performed, followed by 180 min of reperfusion before the rats were sacrificed. The design of the four rat groups in this study is outlined in Figure 1.

### 2.3. Experimental Protocol

To induce anesthesia, rats were intraperitoneally administered Zoletil 50 (tiletamine + zolezepam; Virbac (Taiwan) Co., Ltd. (Taipei, Taiwan)) at a dose of 20 mg/kg body weight and Balanzine (xylazine 2% *w*/*v*) at a dose of 10 mg/kg body weight via intraperitoneal injection [22]. Further, tracheotomy was performed on the rats; subsequently, they were intubated and ventilated. A cannula was then inserted into the carotid artery to allow direct blood pressure (BP) measurement. Biopac MP150 (Biopac Data Acquisition System) was used to directly assess the carotid artery, heart rate, systolic and diastolic BP, and mean arterial pressure (MAP) throughout the experiment. To monitor the cardiac status of each experimental group, hemodynamic changes were detected before, during, and after the experiment. Electrocardiography leads were placed on the limbs of the rats. The proximal section of the left anterior descending coronary artery was encircled with a 4-0 silk suture after median sternotomy. Further, a small vinyl tube was used to create a snare by threading the ends of the silk suture through it. To maintain the body temperature of the rats at 37 °C during the experiments, a rectal thermometer was used to monitor the temperature; moreover, heating pads were used as needed.

After achieving hemodynamic stability for 20 min, rats in the sham group underwent similar surgeries without pretreatment, CAO, or reperfusion. Moreover, no pretreatment was provided to the rats in the CAO group. Conversely, the rats in the HIIT group underwent a 4-week training with HIIT intervention before surgery. Meanwhile, the rats in the IPC group were subjected to two 10 min episodes of CAO to induce IPC, followed by 10 min of reperfusion. Following that, a snare tightened around the left anterior descending coronary artery was used to induce a 40 min coronary artery occlusion in the rats of the CAO, HIIT, and IPC groups. Successful occlusion was confirmed by monitoring the electrocardiogram for ST segment elevation and QRS complex changes and observing cyanotic changes in the occluded area of the myocardium. After 40 min of CAO, the snare was released to allow reperfusion for 3 h. Notably, reperfusion was confirmed by refilling the coronary artery and a reactive hyperemic response. Throughout the experiment, arterial pressure, heart rate, and electrocardiography findings were recorded continuously and simultaneously.

After performing the abovementioned procedure, we obtained blood samples from the rats until euthanasia. The samples were collected from the carotid artery. Further, for biochemical analysis, the serum was separated by centrifugation (2000× *g* at 4 °C for 20 min).

### 2.4. Biochemical Analysis of the Cardiac Function

After the experiments, blood samples of six rats from each group were collected, and serum was separated by centrifugation at 3000 rpm for 15 min. Serum samples were processed in accordance with the manufacturer’s instructions to determine the levels of troponin I (Biovision Incorporated^®^, Milpitas, CA, USA; cat. no. E4737-100) and lactate dehydrogenase (LDH) (LifeSpan Biosciences, Inc.^®^, Seattle, WA, USA; cat. no. LS-F5026).

### 2.5. Histological Examination of Cardiac Injury

After the experiments, the hearts of four rats from each group were collected. Heart tissue was preserved in 10% paraformaldehyde for 24 h at room temperature. After dehydration, the tissue was embedded in paraffin and sectioned into 4 μm sections before being placed on a glass slide. After embedding, the sections were deparaffinized in xylene at room temperature for 10 min and then counterstained with hematoxylin and eosin (HE) at ambient temperature for 7 min. An inverted light microscope (CKX53; Olympus Corporation) was used to analyze the samples at a magnification of 400×. To assess the severity of myocardial injury, a morphological scoring system was used, wherein scores were assigned as follows: 0, no impairment; 1, interstitial edema and focal necrosis; 2, diffuse myocardial cell swelling and necrosis; 3, necrosis with contraction bands and neutrophil infiltrate; and 4, widespread necrosis with contraction bands, neutrophil infiltrate, and hemorrhage. To determine the extent of myocardial damage using HE staining, two examiners independently scored the samples; they were unaware of the sample assignments. The average score was recorded, and the inter-observer agreement was assessed using Pearson’s product-moment correlation coefficient (r = 0.94).

### 2.6. Determination of Area at Risk and MI Size

After the experiments, each group included six rats. Further, the hearts of these rats were collected. Subsequently, an intravenous injection of heparin (1000 U) was administered, followed by resection of the heart and religation of the previous MI site. Further, 1% Evans blue dye (MilliporeSigma, Burlington, MA, USA) was injected into the ascending aorta, and upon completion of perfusion, five transverse sections were obtained from the left ventricle and interventricular septum. Subsequently, these sections were immersed in a solution containing 1% triphenyl tetrazolium chloride and then incubated in a thermostatic water bath at 37 °C for 20 min. After incubation, the sections were weighed and treated with 10% formalin at room temperature for 24 h to ensure fixation. Blue, pale, and red areas corresponded to normal, infarcted, and ischemic tissues, respectively. The total weight of AAR and MI was calculated by adding the weight of myocardial tissue within the vascular territory defined by AAR, located distal to the culprit lesion of the infarct-related artery. AAR and MI were expressed as percentages, with AAR being calculated as the ratio of the weight of the red area to that of the left ventricle and MI being calculated as the ratio of the weight of the white area to that of the red area [23].

### 2.7. Terminal Deoxynucleotidyl Transferase dUTP Nick End Labeling Analysis of the Hearts of Rats

Myocardial cell apoptosis was analyzed by terminal deoxynucleotidyl transferase dUTP nick end labeling (TUNEL) assay using the in situ Cell Death Detection kit, POD purchased from Roche Diagnostics GmbH (Mannheim, Germany; cat. no. 11684817910). After the experiments, the hearts of four rats from each experimental group were sectioned into 4 μm thick slices and incubated in an oven at 68 °C for 1 h and then dewaxed in xylene and gradient alcohol. The sections were then washed three times with phosphate buffered saline (PBS; 5 min each wash). After washing, the sections were placed in hydrogen peroxide and soaked for 10 min. Further, they were washed with PBS, steamed in a pressure cooker for 5 min with citrate buffer at pH 6.0, and washed again three times with PBS (5 min each wash). The sections were then incubated with 50 µL TUNEL reaction mixture (1:12) at 37 °C for 1 h in the dark. Further, they were rinsed three times with PBS, 50 µL POD substrate was added to each section, and the sections were incubated at 37 °C for 30 min in the dark. After incubation, the sections were washed three times with PBS. Subsequently, 50 µL of diaminobenzidine coloring solution was added dropwise, and color development was stopped using distilled water when an appropriate degree of coloration was achieved. The sections were then counterstained with hematoxylin for 2 min, dehydrated in alcohol, and sealed with neutral gum. To determine the final number of TUNEL-positive nuclei, the samples were viewed under an inverted light microscope (CKX53; Olympus Corporation) at a magnification of 400×. Subsequently, we selected a random area and counted the number of nuclei in it. The value was subsequently converted to a percentage by dividing it by the total number of cell nuclei.

### 2.8. Calculation of Serum Levels of TNF-α, IL-1β, IL-6, and IL-10

After the experiments, blood samples were collected from six rats in each group. Enzyme-linked immunosorbent assay (ELISA) was used to measure the serum levels of TNF-α (RTA00), IL-1β (RLB00), IL-6 (R6000), and IL-10 (R1000). The assay was performed using a commercially available ELISA kit (R&D Systems, Inc., Minneapolis, MN, USA) in accordance with the manufacturer’s instructions. Briefly, serum was pipetted into wells precoated with specific antibodies for rat TNF-α, IL-1β, IL-6, or IL-10 and allowed to incubate for 2 h. Further, after the wells were rinsed to remove all unbound substances, an enzyme-linked antibody specific for rat TNF-α, IL-1β, IL-6, and IL-10 was added to the wells for 2 h. After removing the unbound enzyme-linked antibody, the wells were thoroughly washed. Substrate solution was subsequently introduced into the wells and left to react for 30 min, which resulted in the development of a colored product. Optical density readings at 450 nm were then obtained to quantify the intensity of the color. Further, to determine IL-1, IL-6, IL-10, or TNF-α levels in each sample, known standards provided with the kit were used in each assay. Notably, the level of each cytokine was measured in picograms per milliliter. For all kits used in this study, the minimum detectable dose was 10 pg/mL, with intra- and interassay variations of 10%.

### 2.9. Western Blot Analysis of PTEN, Bax-to-Bcl-2 Ratio, TNF-α, Cleaved-Caspase-3-to-Proactive-Caspase-3 Ratio in the Heart

After the experiments, the hearts of four rats from each group were immersed in tissue protein extraction reagent (T-PER; Thermo Fisher Scientific, Inc., Waltham, Massachusetts, MA, USA) at 4 °C to enable homogenization. Further, myocardial cell lysate was prepared by treating the homogenized tissue with a cold lysis buffer comprising 25 mM Tris-HCl (pH 7.6), 150 mM NaCl, 1% NP-40, 1% sodium deoxycholate, and 0.1% sodium dodecyl sulfate. After centrifugation at 10,000× *g* for 10 min at 4 °C, protein levels in the samples were determined using the Bradford assay method. Further, after loading 60 μg of protein onto a 15% sodium dodecyl sulfate polyacrylamide gel, the proteins were separated and transferred to a nitrocellulose membrane. The membrane was blocked with 5% powdered skim milk in Tris-buffered saline comprising 0.1% Tween-20 (TBST) at 37 °C for 30 min and then incubated with PTEN (cat. no. 9583S; Cell signaling), anti-Bax (cat. no. NBP1-28566; Novus Biologicals, Inc., Centennial, CO, USA), anti-Bcl-2 antibodies (cat. no. 633502; BioLegend, San Diego, CA, USA), anti-TNF-α (cat. no. sc-52746; Santa Cruz Biotechnology, Santa Cruz, CA, USA.), and cleaved-caspase-3 (cat. no. IMG-144A; Imgenex, San Diego, CA, USA) at a dilution of 1:1000 in 5% powdered skim milk at 4 °C for 24 h. The nitrocellulose membranes were then immersed in a solution containing a secondary antibody (m-IgGκ BP-HRP; cat. no. sc-516102; Santa Cruz Biotechnology; 1:1000) conjugated to horseradish peroxidase. Subsequently, the membranes were washed with TBST. An enhanced chemiluminescence substrate system was used to visualize peroxidase activity, and the membranes were then exposed to hyperfilms. β-actin (cat. no. 643802; BioLegend; 1:1000) was added as a loading control and incubated at room temperature for 2 h. Densitometry analysis was performed using ImageJ version 1.6 (National Institutes of Health), and normalization against the background density was performed for each gel.

### 2.10. Statistical Analysis

The results were reported as mean ± standard deviation, and statistical analysis was conducted using SPSS version 20.0 (IBM Corp., Armonk, NY, USA). Hemodynamic variables were assessed using two-way repeated measures analysis of variance (ANOVA). Alternatively, the data were evaluated using one-way ANOVA and Bonferroni post hoc test for multiple comparisons. To analyze the ordinal values of the myocardial injury scores, the Mann–Whitney U test was performed with a significance level of *p* < 0.05. Power analysis is a calculation used to determine the minimum sample size for a study. The sample size determination and power analysis of this study were conducted using the G*Power software (Version 3.1.9.7, released on 17 March 2020; *p* = 0.727).

## 3. Results

The differences between hemodynamic variables among the four groups was as follows. At baseline and throughout the experiment, no significant intergroup differences were noted in terms of MAP and heart rate. Furthermore, differences in hemodynamic variables were not significant for all the four groups (See Appendix A Table A1)

### 3.1. Heart Tissue and Serum Analysis

#### 3.1.1. AAR and MI Size Analysis

**Figure 2:**
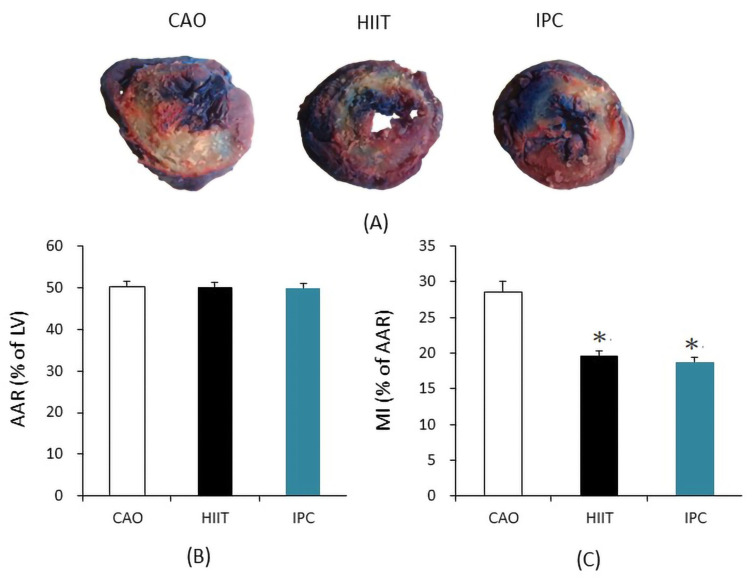
(**A**) Area at risk for ischemia (AAR; Evans blue staining) and infarct area (IFA; 1% 2,3,5-triphenyl-2H-tetrazolium chloride (TTC)) in representative heart sections from the CAO, HIIT, and IPC groups. (**B**) Size of AAR, expressed as a percentage of LV. (**C**) Size of MI, expressed as a percentage of AAR. * *p* < 0.001 vs. CAO group (n = 6). AAR = area at risk; LV = left ventricle; MI = myocardial infarct.

Figure 2A shows the AAR of the left ventricular infarction area. Figure 2B shows the percentage of apoptotic cells in the AAR after staining (MI). The results indicated no significant differences in the AAR between the CAO, HIIT, and IPC groups (50.29 ± 1.14% vs. 50.19 ± 1.18% vs. 49.93 ± 1.08%, respectively, *p* > 0.05). However, MI was significantly reduced in the HIIT and IPC groups compared to the CAO group (19.57 ± 0.67% in HIIT and 18.79 ± 0.60% in IPC vs. 28.57 ± 1.53% in CAO, *p* < 0.001).

#### 3.1.2. Biochemical Analysis of Cardiac Function

**Figure 3:**
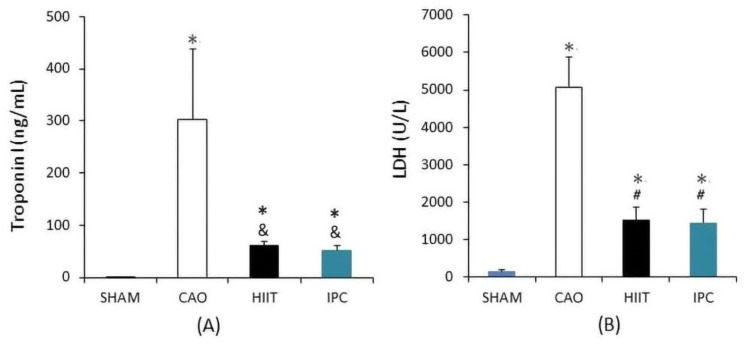
Biochemical analysis of cardiac function. (**A**) Troponin I level. (**B**) Lactate dehydrogenase (LDH) level. *, *p* < 0.001 vs. sham group; &, *p* < 0.01 vs. CAO group; #, *p* < 0.001 vs. CAO group (n = 6). CAO, coronary artery occlusion; LDH, lactate dehydrogenase.

Biochemical analysis was used to assess the function of the heart based on blood samples collected from six rats in each group immediately after the tests. Compared with the sham group, the CAO group had significantly higher serum levels of troponin I (302.64 ± 135.74 vs. 0.55 ± 0.37 ng/mL in the sham group, *p* < 0.001, Figure 3A) and LDH (5066.33 ± 811.77 vs. 143.83 ± 49.91 U/L in the sham group, *p* < 0.001, Figure 3B). The HIIT and IPC groups showed a significantly lower increase in the serum levels of troponin I (61.19 ± 6.76 and 51.81 ± 8.28 ng/mL, respectively, *p* < 0.01 vs. CAO group, Figure 3A) and LDH (1541.33 ± 318.45 and 1450.67 ± 354.84 IU/L, respectively, *p* < 0.001 vs. CAO group, Figure 3B) than the CAO group. However, there were no significant differences between the HIIT and IPC groups in terms of these levels (*p* > 0.05).

#### 3.1.3. Histological Examination of Cardiac Injury

**Figure 4:**
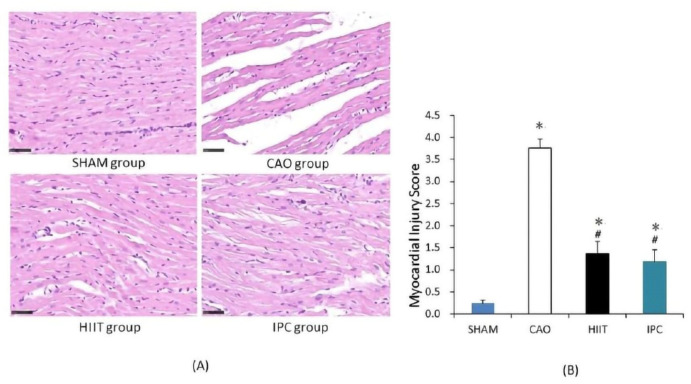
Histological examination of cardiac injury. (**A**) Representative photomicrographs of heart sections stained with hematoxylin and eosin (400× magnification). Normal morphology of the rats’ myocardial tissue in the sham group. Interstitial swelling, myocardial cell edema, and myocardial fibers disruption in the CAO group. (**B**) Histological injury scoring of the myocardial tissue. *, *p* < 0.001 vs. sham group; #, *p* < 0.001 vs. CAO group (n = 4). CAO, coronary artery occlusion.

After the experiments, parts of the hearts of four rats from each group were used for histological evaluation of cardiac injury. In the sham group, the rats exhibited a normal myocardium in the left ventricle (Figure 4A). However, MIRI caused interstitial swelling, myocardial cell edema, and myocardial fiber disturbance. Notably, there was a significant increase in the myocardial injury scores of the CAO group compared with those of the sham group (*p* < 0.001 vs. sham group, Figure 4B). However, HIIT and IPC treatment significantly reduced histological damage. The myocardial injury scores of the rats in both the HIIT and IPC groups appeared to be significantly decreased (*p* < 0.001 vs. CAO group).

#### 3.1.4. TUNEL Staining of Hearts

**Figure 5:**
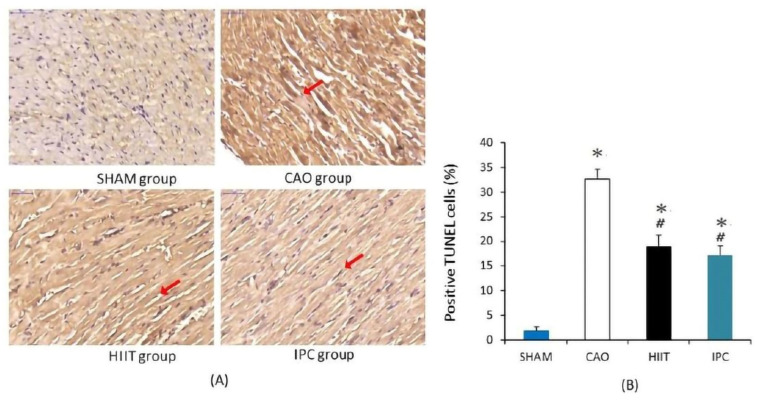
Cardiac apoptosis analysis. (**A**) Illustrative photomicrographs of TUNEL staining (400× magnification). TUNEL-positive nuclei are stained in dark brown and indicated with red arrow. (**B**) The percentage of TUNEL-positive nuclei. *, *p* < 0.001 vs. sham group; #, *p* < 0.001 vs. CAO group (n = 4). TUNEL, terminal deoxynucleotidyl transferase-mediated dUTP nick end labeling.

After the experiments, the hearts of four rats from each group were stained with TUNEL. This staining localized DNA fragmentation in the nuclei of apoptotic cardiomyocytes. The reaction product was dark brown. In the sham group, there were few stained nuclei in the hearts of rats (Figure 5A). In the CAO group, myocardial ischemia–reperfusion induced many TUNEL-positive nuclei in the hearts of rats. Meanwhile, scattered nuclei in the hearts of rats from the HIIT and IPC groups exhibited a dark brown product. There was a significant increase in the number of TUNEL-positive nuclei, presented as a percentage of total nuclei, in the hearts of rats in the CAO group (32.7 ± 1.9% vs. 1.9 ± 0.8% in the sham group, *p* < 0.001, Figure 5B). The HIIT and IPC groups had significantly lower increases in the number of TUNEL-positive nuclei in the heart (19.0 ± 2.2% and 17.2 ± 1.8%, respectively, *p* < 0.001 vs. CAO group, Figure 5B). These groups exhibited no significant differences in terms of TUNEL-positive nuclei (*p* > 0.05) (Figure 5B).

#### 3.1.5. Determination of Serum Levels of TNF-α, IL-1β, IL-6, and IL-10

**Figure 6:**
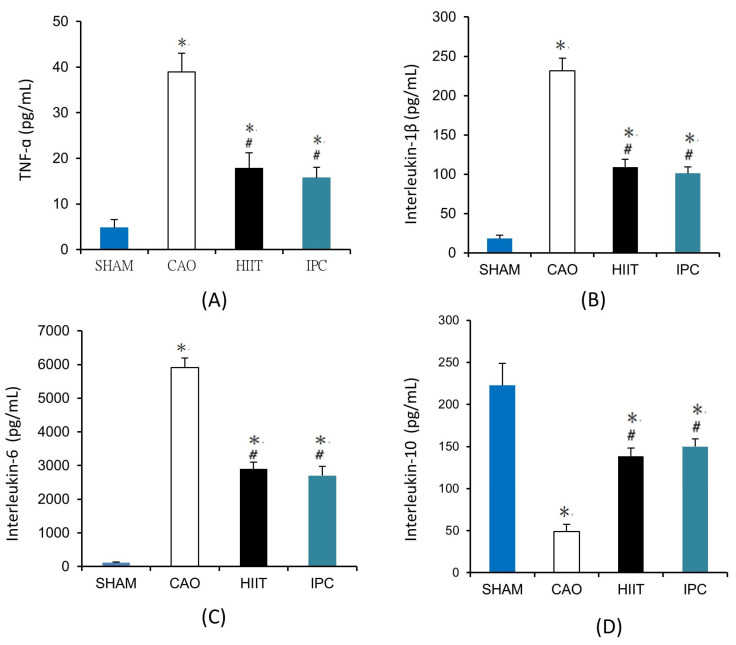
Serum levels of (**A**) tumor necrosis factor-α (TNF-α), (**B**) interleukin-1β, (**C**) interleukin-6, (**D**) interleukin-10. *, *p* < 0.001 vs. sham group; #, *p* < 0.001 vs. CAO group. Blood samples were collected from six rats in each group to determine the serum levels of TNF-α, interleukin-1β, interleukin-6, and interleukin-10 (n = 6). TNF-α, tumor necrosis factor-α.

We evaluated the levels of inflammatory cytokines in serum samples collected from six rats in each group. Myocardial ischemia—reperfusion significantly increased the serum levels of proinflammatory cytokines, including TNF-α (38.96 ± 4.07 vs. 4.77 ± 1.83 pg/mL in the sham group, *p* < 0.001, Figure 6A), IL-1β (231.67 ± 15.90 vs. 18.17 ± 4.37 pg/mL in the sham group, *p* < 0.001, Figure 6B), and IL-6 (5905.45 ± 282.122 vs. 114.45 ± 24.81 pg/mL in the sham group, *p* < 0.001, Figure 6C). However, HIIT and IPC significantly reduced these increases in the serum levels of TNF-α (17.78 ± 3.45 and 15.72 ± 2.32 pg/mL, respectively, *p* < 0.001 vs. CAO group, Figure 6A), IL-1β (109.00 ± 9.86 and 101.21 ± 7.97 pg/mL, respectively, *p* < 0.001 vs. CAO group, Figure 6B), and IL-6 (2900.99 ± 196.77 and 2701.15 ± 280.19, *p* < 0.001 vs. CAO group, Figure 6C). There was a significant decrease in the serum level of the anti-inflammatory cytokine IL-10 due to myocardial ischemia–reperfusion in rats in the CAO group (49.11 ± 8.39 vs. 222.73 ± 26.19 pg/mL in the sham group, *p* < 0.001, Figure 6D). However, HIIT and IPC treatment significantly inhibited the reduction in the serum level of IL-10 (138.36 ± 9.86 and 149.86 ± 9.24 pg/mL, respectively, *p* < 0.001 vs. CAO group, *p* < 0.001, Figure 6D).

#### 3.1.6. Assay of PTEN, Bax-to-Bcl-2 Ratio, TNF-α, and Cleaved-Caspase-3-to-Proactive Caspase-3 Ratio in the Heart

**Figure 7:**
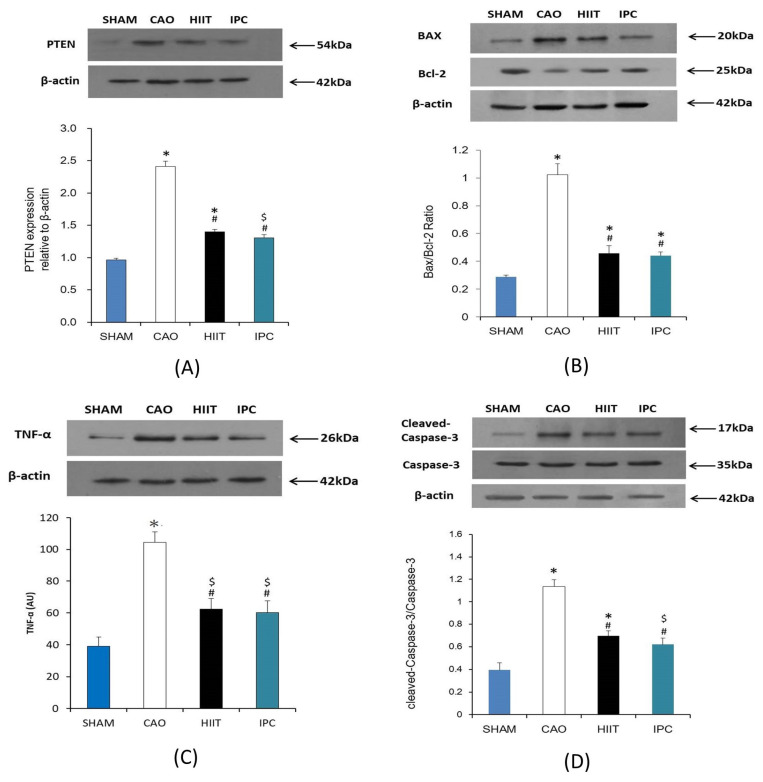
Evaluation of (**A**) PTEN, (**B**) Bax-to-Bcl-2 ratio, (**C**) TNF-α, and (**D**) cleaved-caspase-3-to-proactive-caspase-3 ratio. Illustrative Western blots of PTEN, Bax-to-Bc-2 ratio, TNF-α, cleaved-aspase-3-to-proactive-caspase-3 ratio (upper). The density of PTEN, Bax-to-Bc-2 ratio, TNF-α level, cleaved-caspase-3-to-proactive-caspase-3 ratio were analyzed using arbitrary units (lower). *, *p* < 0.001 vs. sham group; $, *p* < 0.05 vs. sham group; #, *p* < 0.001 vs. CAO group CAO, coronary artery occlusion; TNF-α, tumor necrosis factor-α.

After the experiments, Western blot analysis of PTEN, Bax, Bcl-2, TNF-α, and activated caspase-3 was performed on the hearts of four rats from each study group. The results revealed that myocardial ischemia–reperfusion significantly increased the PTEN levels in the hearts of rats in the CAO group (lane 2, *p* < 0.001 vs. sham group, Figure 7A). Meanwhile, HIIT and IPC significantly reduced the increase in the PTEN levels due to myocardial ischemia–reperfusion (lanes 3 and 4, *p* < 0.001 vs. CAO group, Figure 7A). The Bax-to-Bcl-2 ratio in the hearts of rats from the CAO group was significantly increased due to myocardial ischemia–reperfusion compared with that in the hearts of rats from the sham group. This was confirmed by a significant increase in the density of the Bax band and a decrease in the density of the Bcl-2 band (lane 2, *p* < 0.001 vs. sham group, Figure 7B). HIIT and IPC significantly inhibited the increase in the Bax-to-Bcl-2 ratio (lanes 3 and 4, *p* < 0.001 vs. CAO group, Figure 7B). However, there was no significant change in the Bax-to-Bcl-2 ratio between the HIIT and IPC groups (Figure 7B). Myocardial ischemia–reperfusion significantly increased the TNF-α levels in the hearts of rats in the CAO group (lane 2, *p* < 0.001 vs. sham group, Figure 7C). The HIIT and IPC treatments significantly reduced the increase in TNF-α levels induced by myocardial ischemia–reperfusion (lanes 3 and 4, *p* < 0.001 vs. CAO group, Figure 7C). Western blot analysis revealed that the expression of activated caspase-3 was increased in the heart by myocardial ischemia–reperfusion, as proved by the significantly improved density of the activated caspase-3 band (lane 2, *p* < 0.001 vs. sham group, Figure 7D). Meanwhile, cleaved-caspase-3 was inhibited by HIIT and IPC (lanes 3 and 4, *p* < 0.001 vs. CAO group, Figure 7D). The HIIT and IPC groups did not exhibit any significant differences in the expression of activated caspase-3 (Figure 7D).

## 4. Discussion

The present study proved that 4-week HIIT before MI could significantly decrease MI size, serum levels of MI markers (troponin I and LDH), myocardial tissue swelling and destruction, serum proinflammatory cytokine levels, inflammation, and expression of proapoptosis proteins in rats, thereby decreasing cardiomyocyte apoptosis and exerting cardioprotective effects the same as those associated with IPC intervention. Previous studies have indicated that exercise induces IPC, thereby demonstrating cardioprotective effects through the replication of minor, localized ischemic episodes [24].

The size of the infarct is known as an appropriate indicator of cardiac resistance to IR injury [25]. The results of our study indicated that HIIT and IPC can significantly reduce the MI size after myocardial ischemia–reperfusion, aligning with results previously observed by other scholars [26,27]. However, there was no significant difference in AAR among the three groups; thus, reduced MI size cannot be attributed to surgery-related changes. Cardiac inflammatory response, increased infarct size, and impaired cardiac function are some of the consequences of oxidative stress induced by myocardial ischemia–reperfusion. In the myocardium undergoing ischemia–reperfusion, there is a significant infiltration of neutrophils, characterized by granules containing enzymatically active substances such as gelatinase, collagenase, and elastase. This influx contributes to an exacerbated inflammatory response in the myocardium, leading to increased myocardial damage and expanded infarct size [28]. It is currently known that both IPC and appropriate intensity exercise can induce oxidative stress in the heart, leading to adaptive changes. There are multiple underlying mechanisms, among which includes the activation of protective pathways mediated by reactive oxygen species (ROS) [29,30]. IPC attenuates ROS/Reactive Nitrogen Species (RNS) formation, which may contribute to preserving myocardial oxygen consumption and mitochondrial function, leading to the attenuation of hyperoxygenation after myocardial ischemia and reperfusion [30]. Exercise has been shown to enhance the capacity of the ROS scavenging system and increase the expression of manganese superoxide dismutase (MnSOD) in the mitochondrial matrix. Moreover, exercise can maintain cellular stability by inhibiting apoptosis and decreasing metabolic waste in cells, thereby reducing cardiomyocyte damage after myocardial ischemia–reperfusion injury [31].

In cases of myocardial ischemia–reperfusion, ischemia and hypoxia decrease the mitochondrial matrix, causing expansion of the intermembrane space. This further leads to the destruction of endocardial components in myocardial ischemia sites. In addition, the activity of many enzymes participating in biochemical reactions can decrease or be lost due to myocardial ischemia–reperfusion [32]. In the present study, HE staining of myocardial sections revealed disordered arrangement of myocardial fibers, interstitial expansion, and local cardiomyocyte loss in the CAO group. IPC can activate protein kinase C (PKC) to open mitochondrial ATP-dependent potassium channels (mitoKATP channels), thereby shortening the duration of action potentials, decreasing ATP consumption, myocardial enzyme leakage, and damage caused by free radicals, and ultimately decreasing myocardial ischemia–reperfusion injury [33]. However, HIIT intervention could increase mitochondrial biosynthesis and functions and decrease ATP consumption to decrease energy metabolism in cells. This could significantly decrease the number of myofibroblasts and the content of myocardial interstitial collagen and increase the density of cardiac capillaries, thereby improving cardiomyocyte function, aiding in myocardial repair, and decreasing cardiac injury, which further decreases MI size, cardiac deformation, and enlargement after myocardial ischemia–reperfusion [34,35,36].

In the current study, following myocardial ischemia–reperfusion, the CAO group exhibited elevated levels of blood troponin I and LDH, indicative of myocardial injury. In contrast, both the HIIT and IPC groups demonstrated significantly lower levels of these two enzymes compared to the CAO group, consistent with findings from Ramez et al. [37]. Cardiomyocytes comprise abundant mitochondria, making them particularly vulnerable to electron transport chain damage and increased mitochondrial O_2•−_ generation during ischemia and reperfusion. IPC achieves a reduction in MIRI by accelerating mitochondrial oxidative phosphorylation, decreasing mitochondrial ATP consumption, and enhancing potassium ion efflux. Physical activity, known to exert shear stress and activate potassium channels, enhances mitochondrial ATP synthesis via oxidative phosphorylation. Furthermore, exercise activates the AMP-activated protein kinase (AMPK), a key regulator of metabolism and apoptosis prevention, thereby enhancing peroxisome proliferator-activated receptor gamma coactivator 1-alpha (PGC-1α) to drive metabolic changes and antioxidant responses [33,38,39,40]. Moreover, HIIT has been demonstrated to enhance nitric oxide levels by activating potassium ion channels, leading to increased myocardial vascular endothelial growth factor (VEGF). This promotes angiogenesis, maintaining myocardial cell membrane integrity, reducing the release of MI-related enzymes, and providing superior cardiovascular benefits in physiological remodeling [41,42].

Myocardial ischemia–reperfusion induces ROS synthesis, triggering the release of proinflammatory factors (such as TNF-α, IL-6, IL-1β). Achieving an IL-10/TNF-α equilibrium represents a potential anti-inflammatory treatment strategy for MIRI [43]. Proinflammatory factors induce DNA fragmentation in myocardial cells, elevate the expression levels of pro-apoptotic proteins, such as caspase-3 and Bax, while simultaneously decreasing the expression levels of the anti-apoptotic protein Bcl-2. Both Bax and Bcl-2 have been identified as crucial proteins involved in the formation of mitochondrial apoptotic pathways, regulation of mitochondrial permeability, and transmission of mitochondria-related apoptotic signals [44]. Consequently, this culminates in the generation of cell apoptosis associated with pathological conditions leading to cardiac dysfunction [44,45]. A recent study indicates that IPC enhances ventricular cytochrome c oxidase activity and induces PKC-ε-cytochrome oxidase subunit IV (COIV) coimmunoprecipitation, inhibiting mitochondrial permeability transition pore (mPTP) opening, attenuating calcium overload induced by ischemia reperfusion, and activating the PI3K/AKT signaling pathway. Ultimately, these mechanisms contribute to cardioprotection by reducing oxidative stress, alleviating calcium overload, suppressing cell apoptosis and necrosis, and enhancing mitochondrial dysfunction in the face of potentially fatal ischemia and reperfusion injury [46,47]. HIIT intervention in rats with IR injury has been shown to boost angiogenic factors, including VEGF and FGF2, influencing apoptosis through PI3K/Akt-mediated regulation of antiapoptotic proteins (such as BCL-2, survivin, and XIAP) and elevated nitric oxide levels [41,48]. Moreover, HIIT intervention can downregulate the expression of the angiostatic factor Thrombospondin-1 (TSP-1) in rats with IR injury, which in turn can activate caspase-3 and promote apoptosis through endothelial nitric oxide synthase dephosphorylation, BCL-2 and BCL-XL depletion, and activation of the p38 group of mitogen-activated protein kinases [42].

In this study, we found a significant increase in the PTEN levels within the myocardial tissue after HIIT intervention and IPC. Notably, the mitochondrial levels of PTEN and Bax were increased in rats with IR injury. Reperfusion after ischemia causes a burst of ROS, including hydrogen peroxide (H_2_O_2_), increasing the mitochondrial localization of PTEN in isolated hearts [49]. Increasing evidence suggests that PTEN inactivation is associated with reduced apoptosis. The decreased ROS production induced by IPC may mitigate the localization of PTEN in mitochondria, resulting in reduced Bax translocation and apoptosis [49]. After exercise training, MicroRNA-486 (MiR-486) is upregulated in the heart. This microRNA directly targets and negatively regulates PTEN, thereby activating the AKT/mTOR pathways to protect cardiomyocytes against apoptosis. Furthermore, it prevents apoptosis induced by H_2_O_2_ and coronary microembolization in the cardiovascular system. Additionally, MiR-486 plays a pivotal role in mediating the protective effect of circulating extracellular vesicles against cardiomyocyte apoptosis [50].

This study had some limitations. First, one of the primary factors affecting the size of MI is the extent of the AAR and the degree of collateral circulation to the coronary vessels; however, we found no significant intergroup differences in the AAR size, which was expressed as a percentage of left ventricular weight. Second, owing to methodological constraints, we could not obtain definitive data on myocardial blood flow. Nevertheless, our model, which was similar to the models widely used in studies on myocardial ischemia and preconditioning, was appropriate given the limited collateral coronary circulation observed in rats [51]. Third, TUNEL staining is a sensitive method; however, it is not specific for detecting cell apoptosis and might result in the staining of noncardiomyocyte DNA fragmentation. In our study, we also performed Western blot analysis, which provides a specific and quantitative analysis of apoptosis. 

## 5. Conclusions

In conclusion, this study was the first to simultaneously compare the effects of IPC and HIIT on myocardial ischemia–reperfusion. HIIT intervention reduced myocardial infarct size, decreased serum concentrations of troponin-I and LDH associated with myocardial infarction, and also reduced serum levels of TNF-α, IL-1β, and IL-6 while increasing the anti-inflammatory cytokine IL-10, demonstrating similar myocardial protective effects as IPC. Both interventions could decrease the expression of pro-apoptotic proteins PTEN, TNF-α, caspase-3, and Bax, while increasing the expression of anti-apoptotic protein Bcl-2, thereby reducing inflammation and myocardial cell apoptosis. In human applications, IPC provides a potent endogenous cardioprotective strategy. It reduces the size of MI in patients with ST-segment elevation myocardial infarction (STEMI) undergoing reperfusion. Moreover, it attenuates perioperative and periprocedural myocardial injury in patients undergoing coronary artery bypass graft (CABG) surgery or percutaneous coronary intervention (PCI). However, exercise training is a clinically validated primary intervention with “cost-effectiveness”, proven to “delay” and even “prevent” the health burden associated with numerous chronic diseases. HIIT seems safe for patients with cardiovascular disease, such as coronary artery disease and heart failure, in a tertiary-care cardiac rehabilitation setting, and its role within this setting warrants further consideration given the robust evidence of its efficacy in enhancing various cardiovascular health and fitness measures. The findings of this study provide medical insights into the prevention and reduction of myocardial injury through IPC and HIIT interventions. Considering primary and secondary disease prevention strategies, and enhancing physical function through exercise may be one of the simplest and most cost-effective methods to promote cardiovascular health.

## Figures and Tables

**Table 1:** Detailed description of the HIIT group exercise protocol.

		Rat Age	Wk3	Wk4	Wk5	Wk6	Wk7	Wk8
Variable/Measure								
Max. speed	meters/min		20	30	50	50	50	50
Incline	degrees		5	10	10	10	10	10
Duration	minutes		30	30	30	30	30	30
Intensity	2 min stationary period followed by 1 min sprint period × 10 reps							
Frequency	bouts/wk		4	4	4	4	4	4
Distance per bout	meters		200	300	500	500	500	500
Distance per week	meters		800	1200	2000	2000	2000	2000

## Data Availability

The data that support the findings of this study are available from the corresponding author, CCL, upon reasonable request.

## Appendix A

**Table A1:** Hemodynamic changes during the experiments ^a^.

	Group	Baseline	CAO		CAR		
			20 min	40 min	1 h	2 h	3 h
Mean arterial pressure, mmHg							
1	SHAM	80 ± 3	79 ± 9	79 ± 3	81 ± 8	82 ± 4	81 ± 3
2	CAO	83 ± 8	75 ± 4	74 ± 4	75 ± 6	78 ± 5	80 ± 4
3	HIIT	81 ± 6	77 ± 7	75 ± 5	76 ± 4	78 ± 7	82 ± 5
4	IPC	82 ± 4	78 ± 5	76 ± 2	76 ± 3	79 ± 2	83 ± 6
Heart rate, beats/min							
1	SHAM	431 ± 27	427 ± 22	431 ± 33	435 ± 31	440 ± 19	438 ± 21
2	CAO	437 ± 29	460 ± 28	456 ± 27	451 ± 19	448 ± 26	450 ± 18
3	HIIT	451 ± 18	453 ± 31	440 ± 24	446 ± 23	446 ± 37	449 ± 33
4	IPC	442 ± 30	454 ± 39	455 ± 17	452 ± 18	451 ± 40	452 ± 26
Mean arterial pressure-heart rate product/1000, mmHg×eats/min							
1	SHAM	35.26 ± 2.17	35.26 ± 1.94	34.40 ± 2.38	34.18 ± 2.01	34.47 ± 1.38	35.01 ± 2.03
2	CAO	38.67 ± 1.72	37.35 ± 1.29	36.04 ± 1.88	37.75 ± 2.11	37.82 ± 1.84	38.28 ± 2.35
3	HIIT	36.48 ± 2.38	34.85 ± 1.24	34.58 ± 1.22	35.46 ± 1.38	34.19 ± 2.11	35.21 ± 1.48
4	IPC	37.29 ± 1.21	35.37 ± 1.44	35.04 ± 2.50	36.00 ± 2.54	36.89 ± 1.68	37.08 ± 2.33

^a^ Data are presented as mean ± standard deviation. Baseline = baseline before treatment; CAO = coronary artery occlusion; CAR = coronary artery reperfusion; HIIT = HIIT training group; IPC = ischemic preconditioning group. Rats in the sham group did not receive any pretreatment, coronary artery occlusion, or reperfusion.
